# *Proteus mirabilis* exacerbates ulcerative colitis by inhibiting mucin production

**DOI:** 10.3389/fmicb.2025.1556953

**Published:** 2025-03-25

**Authors:** Zhihui Jiang, Pengpeng Li, Kehui Qiu, Yang Liao, Xin Chen, Ji Xuan, Fangyu Wang, Hongfeng Ma, Ye Wang, Minsheng Zhu

**Affiliations:** ^1^State Key Laboratory of Pharmaceutical Biotechnology, Suqian Scientific Research Institute of Nanjing University Medical School, Gulou Hospital of the Medical School, Nanjing University, Nanjing, China; ^2^Department of Gastroenterology, Jinling Hospital, The Medical School of Nanjing University, Nanjing, China; ^3^Department of Rehabilitation Medicine, Huzhou Rehabilitation Hospital, Huzhou, China

**Keywords:** ulcerative colitis, *Proteus mirabilis*, mucus layer, IL-18, bacteriophage

## Abstract

**Introduction:**

Ulcerative colitis (UC) is characterized by chronic inflammation and ulceration in colonic mucosa, accompanied by a defective epithelial barrier. *Proteus mirabilis* (*P. mirabilis*) bacterium is a putative intestinal pathogen with invasive ability, yet its role in UC inflammation and gut barrier disruption is unclear. This study aims to investigate its epidemiological presence, pathogenic roles and preventive strategy during UC inflammation.

**Method:**

*P. mirabilis* culture and PCR amplification of the *P. mirabilis*-specific *ureR* gene were used to detect fecal P. mirabilis and determine its prevalence in UC and control stool specimens. *P. mirabilis* isolated from UC stool specimens was gavaged into dextran sulfate sodium (DSS)-treated mice. Inflammation and the mucus layer of colons were assessed through histological examination and cytokine quantification. Bacteriophages were screened and used to eliminate *P. mirabilis* in colitis animals.

**Results and discussion:**

The fecal *P. mirabilis* bacteria were detected by PCR amplification of *P. mirabilis*-specific *ureR* gene. Of 41 UC patients, 65.9% patients were *P. mirabilis* positive, which was significantly higher than the controls. Administration of *P. mirabilis* aggravated DSS-induced colitis symptom and mucosal inflammation in mice. Interestingly, the colonic mucus layer, an essential component of the epithelial barrier, of the animals was dramatically disrupted, which was consistent with the alteration of human UC colon. The disrupted mucus layer was mediated by the down-regulation of IL-18 in intestinal epithelium. Importantly, a bacteriophage cocktail targeting *P. mirabilis* could restore the mucus barrier and alleviate the enteric inflammation. Thus, our results suggest that *P. mirabilis* is a UC pathobiont bacterium, which exacerbates the severity of UC inflammation owing to down-regulation of mucin production and IL-18 expression. Bacteriophage-mediated elimination of *P. mirabilis* may be effective in limiting UC inflammation.

## 1 Introduction

Ulcerative colitis (UC) is a chronic disease with no definitive therapy due to its unknown etiology. The clinical manifestations of UC primarily include bloody diarrhea and recurrent episodes, often accompanied by extraintestinal complications. In advanced stages, UC may also lead to the development of colorectal cancer (Ungaro et al., 2017). Histologically, UC is characterized by superficial mucosal ulcerations extending proximally from the rectum, disrupting epithelial integrity and barrier function. Factors such as genetic predisposition, environmental influences, gut microbiota dysbiosis, and mucosal immunity dysregulation have been implicated in UC pathogenesis (Chang, 2020; Rogler et al., 2021). Current UC therapies, including aminosalicylates, biologics, and JAK inhibitors, focus on suppressing immune activation but fail to address underlying microbial or mucosal dysfunction. These treatments often yield variable efficacy, with risks of resistance, side effects (e.g., infections, malignancy), and high costs (Baumgart and Le Berre, 2021). Approximately 25% of severe cases necessitate subtotal colectomy, which heavily threaten the life quality of patients (Kobayashi et al., 2020). Thus, exploration of more effective treatments is highly necessary.

A hallmark of UC is intestinal microbial dysbiosis, characterized by reduced diversity and the expansion of pro-inflammatory taxa such as *Gammaproteobacteria, Enterobacteriaceae*, and sulfate-reducing *Deltaproteobacteria* (Frank et al., 2007). These population shift has been considered to be associated with mucosal inflammation and barrier dysfunction, although the causal mechanisms linking specific microbes to UC progression remain elusive (Hendrickson et al., 2002). Among these bacterial populations, *Proteus mirabilis* (*P. mirabilis*), a γ-*Proteobacteria* pathobiont, may exacerbate the process of gut inflammation in Crohn's disease (CD) and post-surgical recurrence (Mondot et al., 2016; Wright et al., 2017). However, its role in UC inflammation remains unknown.

*P. mirabilis* is a gram-negative bacterium belonging to the *Enterobacteriaceae* family and is a member of the gastrointestinal microbiota. Due to its low abundance in healthy gut microbiota, *P. mirabilis* is rarely detected in the feces of healthy individuals (Zhang et al., 2021). Under conditions of enteric inflammation, however, *Proteus* spp. along with and nitrate-reducing *Enterobacteriaceae* may be expanded under anaerobic conditions by elevated nitrate produced by inducible nitric oxide synthase (iNOS) (Winter et al., 2013; Xia et al., 2022). It has been reported that *P. mirabilis* exacerbates gut inflammation by inducing IL-1β expression and *Klebsiella pneumoniae* may synergize this process (Garrett et al., 2010; Seo et al., 2015; Hamilton et al., 2018; Zhang et al., 2021). Thus, *P. mirabilis* potentially contributes to UC-associated barrier disruption and immune dysregulation.

*P. mirabilis* has been observed to translocate to mesenteric lymph nodes and liver of the mice compromised gut microbiota (Wells and Erlandsen, 1991; Nakamoto et al., 2019). These observations imply that *P. mirabilis* has a capacity to disrupt intestinal mucosal barrier that usually comprising epithelial monolayer, subepithelial cells and mucus layer. As active UC is often characterized by depletion of colonic mucin-producing goblet cells and a compromised mucus layer (Strugala et al., 2008), we here hypothesized that *P. mirabilis* exacerbated UC pathology through targeting the mucus barrier. To address this, we used bacterial culture and PCR amplification methods to detect the presence of fecal *P. mirabilis* from UC patients and control populations, analyzed their prevalence. We then evaluated the role of *P. mirabilis* in colitis and investigated the mechanism underlying. Our results showed a much more frequent presence of *P. mirabilis* in UC patients, and this bacteria aggravated enteric inflammation by inhibiting mucus production via IL-18 expression. Moreover, elimination of *P. mirabilis* by specific bacteriophages can significantly attenuate colitis symptoms. We thus suggest that *P. mirabilis* is an important pathobiont in UC progression and elimination of this bacteria may inhibit the severity of colitis progression.

## 2 Materials and methods

### 2.1 Animal studies

C57BL/6J mice were purchased from GemPharmatech (Jiangsu, China). The animals were housed in SPF animal rooms of the Model Animal Research Center of Nanjing University. Mice were kept under a 12:12 h light:dark cycle and provided with water and food *ad libitum*. All animal procedures in this study were conducted in accordance with the guidelines of the Institutional Animal Care and Use Committee (IACUC) of the Model Animal Research Center of Nanjing University (Nanjing, China).

### 2.2 Human fecal bacteria collection and culture

Stool specimens from 41 UC patients and 32 constipation patients were collected while they were hospitalized in the Department of Gastroenterology at Jinling Hospital. Additionally, healthy stool specimens were collected from 61 healthy volunteers. Participant information is provided in Tables 1, 2. Fresh stool specimens were collected with a collection kit (ChuTianShu Biotechnology Company, Xi'an, China).

**Table 1:** Baseline demographic characteristics of the subjects and *P. mirabilis* detection in stool specimens.

**Group**	**Total *N***	**M/F**	**Age**			
			** < 20**	**20–39**	**40–59**	**≥60**
UC patients	41	22/19	7	12	15	7
*P. mirabilis* positive *N* (%)	27 (65.9%)	13 (59.1%)/14 (73.7%)	5 (71.4%)	8 (66.7%)	9 (60%)	5 (71%)
Healthy population	61	30/31	3	28	13	15
*P. mirabilis* positive *N* (%)	2 (3.3%)^**^	2 (6.7%)/0 (0%)	0 (0%)	0 (0%)	2 (15.4%)	0 (0%)
IFC patients	32	7/25	1	12	17	2
*P. mirabilis* positive *N* (%)	2 (6.3%)^**^	0 (0%)/2(8%)	0 (0%)	0 (0%)	2 (11.8%)	0 (0%)

Chi-square tests were used to compare the *P. mirabilis*-positive percentage of the UC group with the healthy or IFC group.

^**^P < 0.01.

**Table 2:** Disease characteristics of the UC patients participated in *P. mirabilis* epidemiology studies.

	***N* = 41 (%)**
**Extent of disease**	
E1 proctitis	2 (4.9%)
E2 left-sided colitis	12 (29.2%)
E3 pancolitis	27 (65.9%)
**Treatment**	
Aminosalicylates	40 (97.6%)
Corticosteroid	27(65.9%)
Immunosuppressants	8 (19.5%)
Vedolizumab	0 (0%)
Infliximab	2 (4.9%)
**Disease activity**	
Mayo score, MES (mean and range)^a^	2.4 ± 0.6 (0–3)
CRP (mg/dl)^a^	29.3 ± 50.6
Alb (g/dl)^a^	35.6 ± 8.7
ESR (mm/h)^a^	21.0 ± 20.6

^a^values represent mean ± SD. CRP, C-reactive protein; Alb, albumin; ESR, erythrocyte sedimentation rate.

### 2.3 *P. mirabilis* detection with PCR method

UreR is the transcriptional activator of urease gene and is required for urease activity. Since *ureR* is conserved in *P. mirabilis*, detecting the *ureR* gene by PCR method can rapidly and effectively identify the presence of *P. mirabilis* bacteria in stool specimens. A specific primer set targeting *ureR*-gene was used to detect *P. mirabilis* in stool specimens (Jijuan et al., 2008). Briefly, 100 μl of stool specimen suspension was added to 0.9 ml LB medium and cultured for 12–16 h at 37°C, and then subjected to PCR amplification. PCR reaction included 10 μl Taq Master Mix (P112, Vazyme), 1 μl of bacterial suspension, 0.5 μl forward primer (5′-CAACGTGAGATTAGTGGTGA-3′), 0.5 μl reverse primer (5′-CTGCTTATAAGTTCACAAATTAAGTG-3′), 8 μl ddH_2_O. The PCR program was set as follows: (1) pre-incubation at 95°C for 3 min; (2) 35 cycles of denaturation at 95°C for 30 s, annealing at 58°C for 30 s and elongation at 72°C for 30 s; (3) final elongation at 72°C for 5 min. PCR products were separated by agarose gel electrophoresis and the target band was 241 bp.

### 2.4 16s rRNA sequencing and phylogenetic tree

*P. mirabilis* colonies were isolated from stool samples of UC patients. Briefly, UC fecal bacteria were spread on sheep blood agar plates and candidate colonies were picked and identified by *P. mirabilis* specific primer set as described. The 16S rRNA sequence of candidate *P. mirabilis* colonies were amplified and sequenced using the primer set: bacteria-27-forward primer (5′-AGAGTTTGATCCTGGCTCAG-3′) and bacteria-1492-reverse primer (5′-GGTTACCTTGTTACGACTT-3′). The evolutionary history was inferred using the Neighbor-Joining method. The evolutionary distances were computed using the Maximum Composite Likelihood method and are in the units of the number of base substitutions per site. The analysis involved 35 nucleotide sequences. All positions containing gaps and missing data were eliminated. There were a total of 1,301 positions in the final dataset. Evolutionary analyses were conducted using MEGA7.

### 2.5 Histology and immunohistochemistry staining

Mouse colon tissues was fixed with 4% PFA and embedded in paraffin (39601095, Leica). Hematoxylin and eosin (H&E) staining was performed according to standard protocols. Immunohistochemistry was conducted according to the manufacturer's instructions (KIT-9706, MXB Biotechnologies). Briefly, 5 μm paraffin sections of colon tissue were antigen retrieved with citrate buffer (E673001, BBI). After peroxidase treatment and blocking, anti-Muc2 (27675-1-AP, Proteintech) primary antibodies were applied to the sections and incubated at 4°C overnight. Horseradish peroxidase conjugated secondary antibody was further applied to slides. Development was performed using a DAB Peroxidase (HRP) substrate kit (DAB-0031, MXB Biotechnologies), followed by counterstaining with hematoxylin.

### 2.6 PAS-AB staining and mucus layer thickness measurement

To measure the mucus layer, tissue from mice distal colon were fixed with methacarn solution (131291, TIANDZ) at 4°C for 2 h. Fixed tissues were gradually dehydrated with methanol, ethanol, and xylene, and then embedded in paraffin. PAS-AB staining (R20530, Shanghai yuanye) was then performed as manufacturer's instructions using 5 μm paraffin sections of methacarn-fixed tissue. Mucus layer thickness was measured from PAS-AB stained sections. Ten measurements of the mucus layer were taken from each mouse's distal colon tissue, and data are presented as the mean of the 10 measurements.

### 2.7 DSS colitis induction and phenotypic evaluation

To generate acute colitis, male C57BL/6J mice (8 weeks old) were given 2% dextran sodium sulfate (DSS) in water for 7 days, followed by 2 days of regular drinking water. DSS-treated mice received a gavage of 1 × 10^9^ CFU *P. mirabilis* (*P. mirabilis* group, *n* = 12) or 100 μl PBS (PBS group, *n* = 12) on the fifth and sixth day after DSS administration. For phage cocktail treatment, mice were orally administered 2 × 10^8^ PFU *P. mirabilis* targeting phage cocktail (Phage group, *n* = 10) or 200 μl of vehicle control SM phage buffer (Ctr group, *n* = 10) for 3 consecutive days starting on the sixth day after DSS administration. Body weight and disease activity index (DAI) scores were recorded daily. DAI scores included the following: body weight loss (none = 0, < 5% = 1, 5%−10% = 2, 10%−20% = 3, >20% = 4), stool consistency (normal = 0, loose = 2, diarrhea = 4), bleeding (none = 0, occult blood = 2, gross bleeding = 4) (Wirtz et al., 2017). The pathological scores were calculated using the method described in Wirtz et al. (2017): tissue damage (0 = none, 1 = isolated focal epithelial damage, 2 = mucosal erosions and ulcerations, 3 = extensive damage deep into the bowel wall); Lamina propria inflammatory cell (0 = Infrequent, 1 = Increased, some neutrophils, 2 = Submucosal presence of inflammatory cell clusters, 3 = Transmural cell infiltrations). Tissue damage scores and lamina propria inflammatory cell scores were summed to yield the total pathology score.

### 2.8 mRNA quantification by real-time PCR

Total RNA from the distal colon was extracted using FastPure Complex Tissue/Cell Total RNA Isolation Kit (RC113, Vazyme), and reverse transcribed with HiScript III RT SuperMix (R323, Vazyme). The cDNA samples were subjected to a real-time quantitative PCR (qPCR) to measure the mRNA levels of *ifng, tnfa, il6, il17a, il23a, il22, il1b, il12b, ccl2, cxcl1, gapdh, gfi1, spdef*, *klf4, regIII*γ, *relm*β, *muc2, clca1, il18*, and *il18bp*. The qPCR reaction was performed as follows: 5 μl Taq Pro Universal SYBR qPCR Master Mix (Q712-02, Vazyme) along with 4.6 μl of each oligonucleotide with 0.2 μl/0.2 μl forward and reverse primer sets (Genscript) (primers are listed in Supplementary Table 1). The amplification program was set as follows: (1) pre-incubation at 95°C for 30 s; (2) 40 cycles of denaturation at 95°C for 10 s and annealing at 60°C for 20 s on an ABI ViiA™ 7 Real-Time PCR System. Raw data were analyzed using the ΔCt method, with *Gapdh* serving as the normalization control. Relative expression levels were calculated using the 2^−ΔΔCt^ formula. The relative mRNA levels were represented as fold change.

### 2.9 Western blot

Frozen colon tissues were homogenized and lysed in RIPA buffer (50 mm Tris-HCl, pH 7.5, 150 mm NaCl, 1 mm EDTA, 1% NP-40, 0.25% sodium deoxycholate) containing 1 × protease inhibitor cocktail (HY-K0011, MedChemExpress) on ice for 30 min. The samples were then denatured in 1 × Laemmli buffer at 95°C for 5 min, followed by separation using SDS-PAGE and transfer to PVDF membranes (Bio-rad). The following primary antibodies were used: anti-Muc2 (1:5,000, 27675-1-AP, Proteintech), anti-IL-18 (1:5,000, 10663-1-AP, Proteintech), anti-IL-18BP (1:1,000, 14153-1-AP, Proteintech), anti-β-actin (1:5,000, A5441, Sigma-Aldrich). HRP-linked goat anti-mouse IgG (H + L) (1:5,000, #31430, Thermo Fisher) and HRP-linked goat anti-rabbit IgG (H + L) (1:5,000, #31460, Thermo Fisher) were used as secondary antibodies. After incubation with the secondary antibodies for 2 h at room temperature, the membranes were developed using High-sig ECL Western Blotting Substrate (180-5001, Tanon) on a western blotting detection system (Tanon 4160).

### 2.10 Bacteriophage isolation and PFU measurement

Four lytic bacteriophages targeting *P. mirabilis* were isolated from surface water in Nanjing. Briefly, 25 ml of water was mixed with 25 ml of 2 × concentrated LB broth and 1 ml of a 16-h culture *P. mirabilis*. After 24 h incubation at 37°C, with shaking at 200 rpm, the supernatant was syringe-filtered. Lytic bacteriophages were then screened by the double agar overlay method (Kropinski et al., 2009). Individual bacteriophage plaques were picked up with pipette tip for further purification. Bacteriophages were then stored in SM buffer (5.8 g/l NaCl, 2 g/l MgSO_4_, 0.1 g/l gelatin, 50 mm Tris-HCl, pH 7.5). The plaque-forming unit (PFU) of the bacteriophages was measured by the double agar overlay method.

### 2.11 Statistics

Data are presented as mean ± SEM. Statistical significance between two groups was determined using an unpaired Student's *t*-test. The statistical significance (*p*-values) for body weight change was assessed by ordinary two-way *ANOVA*. Chi-square tests were used to compare the *P. mirabilis*-positive percentage of the UC group with the healthy or IFC group. All statistical analyses were conducted using GraphPad Prism 8. Statistical significance values are indicated in each figure legend as follows: n.s. = *P* > 0.05, ^*^P < 0.05, ^**^P < 0.01.

## 3 Results

### 3.1 *P. mirabilis* was frequently detected in UC stool specimens

**Figure 1:**
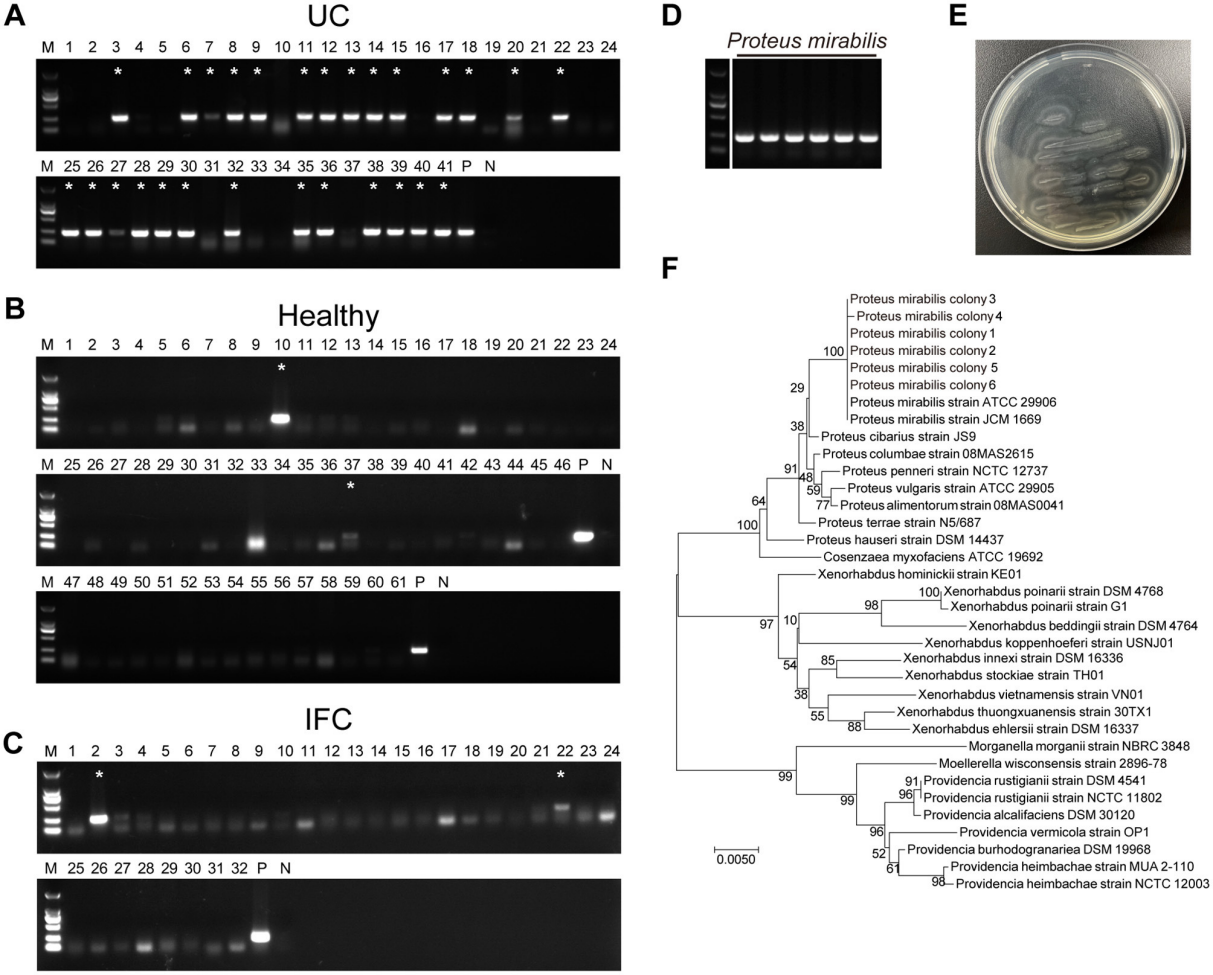
*P. mirabilis* was frequently detected in UC stool specimen. Specific PCR primer set targeting *ureR* gene of *P. mirabilis* was used to identify the epidemiological distribution in stool specimen from **(A)** ulcerative colitis patients, **(B)** healthy volunteers and **(C)** intractable functional constipation patients. **(D)** *P. mirabilis* colonies isolated from UC stool specimens. **(A**–**C)** Asterisks indicate *P. mirabilis-*positive in stool specimens. P, positive control; N, Negative control. **(E)** *P. mirabilis* colonies showed bull-eye pattern and swarming phenotype on LB agar. **(F)** 16S rRNA sequence phylogenetic tree of isolated *P. mirabilis* colonies. The optimal tree with the sum of branch length = 0.21781896 is shown. The tree is drawn to scale, with branch lengths in the same units as those of the evolutionary distances used to infer the phylogenetic tree.

To investigate the distribution of *P. mirabilis* in UC patients, we collected stool samples from 41 UC patients, 61 healthy volunteers, and 32 IFC (intractable functional constipation) patients. UC patients were diagnosed based on the clinical manifestations, endoscopy, and histology, in accordance with established guidelines (Inflammatory Bowel Disease Group et al., 2018), while IFC patients were diagnosed using the Rome III or IV criteria. The clinical characteristics of the participants are summarized in Tables 1, 2. IFC has been reported to exhibit changes in the gut microbiome, but without inflammation or mucosal lesions (Pan et al., 2022; Wu et al., 2024). The purpose of inclusion of IFC stool specimens is to set a disease control which has no mucosal lesion. Considering urease-producing bacteria featured IBD dysbiosis and worse immune-mediated colitis (Ni et al., 2017; Ryvchin et al., 2021), and UreR, the conserved transcriptional activator of urease gene in *P. mirabilis*, is required for urease activity (Dattelbaum et al., 2003; Fitzgerald et al., 2024), we used a specific PCR primer set targeting the *ureR* gene to detect fecal *P. mirabilis*. Among the 41 UC patients, 27 (65.9%) were positive for *P. mirabilis* in their feces, compared to 2 (3.3%) of the 61 healthy controls and 2 (6.3%) of the 32 IFC patients. The prevalence of *P. mirabilis* in UC patients was significantly higher than in the control groups (65.9% vs. 3.3% in healthy volunteers and 6.3% in IFC patients; all *P* < 0.01; Figures 1A–C and Table 1). *P. mirabilis* was rarely detected in IFC patients, suggesting that an inflammatory environment may be associated with a high abundance of *P. mirabilis*. We included UC patients aged 14 to 68 years, and divided them into four age groups (Table 1). There was no significant difference in the prevalence of *P. mirabilis* across age groups and genders in UC patients (all *P* > 0.05). Interestingly, among the healthy participants and IFC patients, *P. mirabilis*-positive specimens were primarily detected in the adults with age from 40 to 59 years. We don't know the reason for this phenomenon. Collective observations indicate a frequent presence of *P. mirabilis* in UC in contrast to the controls, implying a role in UC pathology.

###  3.2 *UreR* gene positive isolates recovered from the UC samples were *P. mirabilis*

Six *ureR* gene positive colonies were recovered from clinical UC fecal samples and identified as candidate isolates of *P. mirabilis* (Figure 1D). All the isolated colonies exhibited typical characteristic of *P. mirabilis* including bull's-eye pattern and swarming motility on LB agar (Figure 1E). The 16S rRNA sequences of these six isolates were amplified and sequenced for phylogenetic analysis. A phylogenetic tree of 16S rRNA showed that all the candidates were identical to ATCC29906 (Gene ID: NR_114419), the standard strain of *P. mirabilis* (Figure 1F). The *P. mirabilis* colonies isolated from UC fecal specimens were used for our further studies.

### 3.3 *P. mirabilis* aggravated colonic inflammation in DSS colitis mice

**Figure 2:**
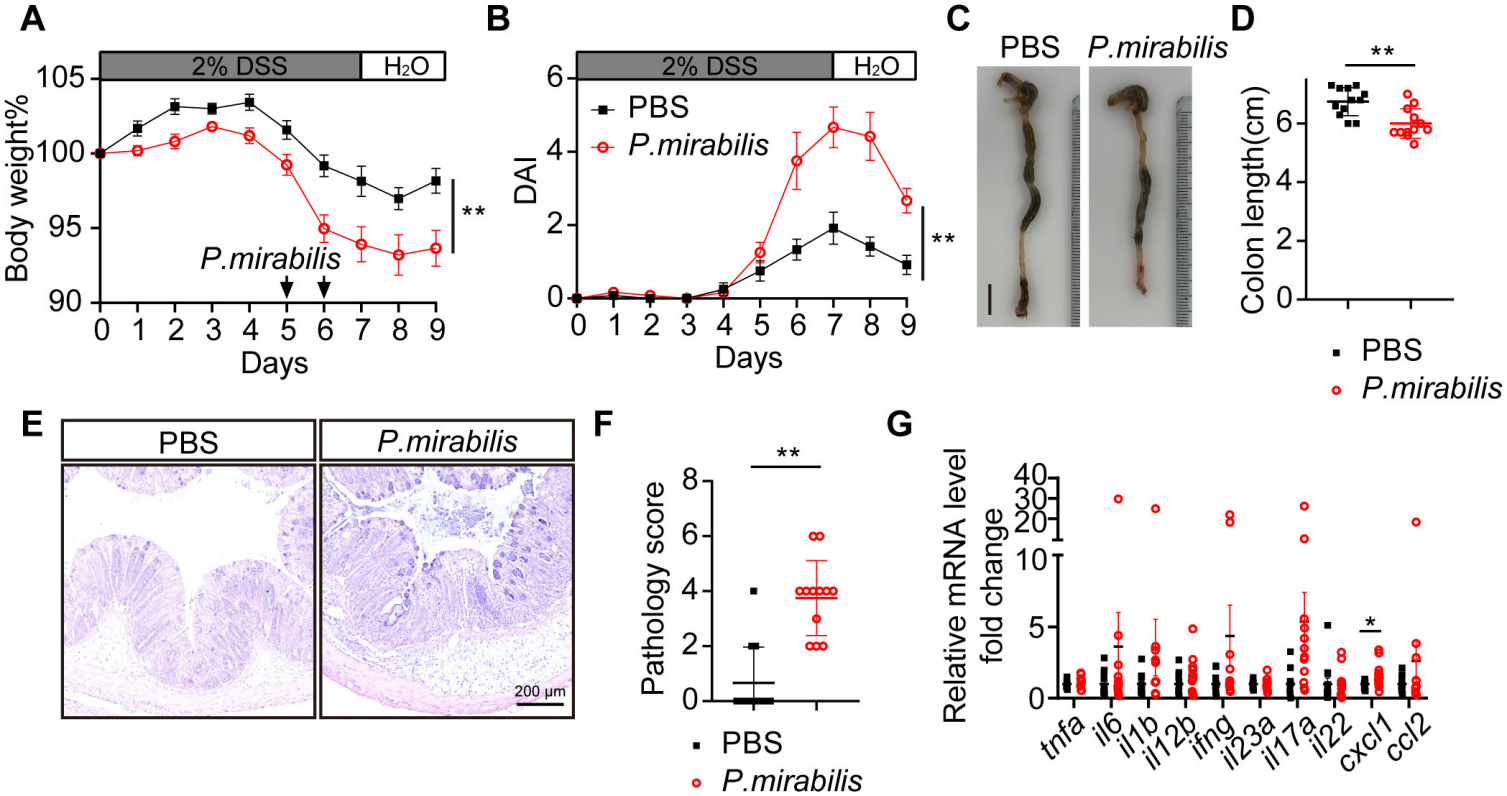
*P. mirabilis* infection during DSS treatment aggravated colitis symptom. Acute colitis was induced with 2% DSS for 7 consecutive days in C57BL/6J mice. Mice received twice gavage of 1 × 10^9^ CFU of *P. mirabilis* bacterium (*n* = 12) or 100 μl PBS (*n* = 12) at black arrow indicated time. **(A)** Body weight change. **(B)** Daily disease activity index. **(C)** Representative picture of colon. Scale bar, 1 cm. **(D)** Colon length. **(E)** H&E staining of colon section. Scale bar, 200 μm. **(F)** Pathology score of colons. **(G)** Relative cytokine mRNA levels of colon, normalized by PBS group. **(A, B, D, F, G)** Data are presented as mean ± SEM. *P < 0.05, **P < 0.01. **(A, B)** Two-way ANOVA with Geisser-Greenhouse correction. **(D, F, G)** Unpaired 2-tailed *t* test. Black square, *P. mirabilis* group; red circle, PBS group.

To assess the role of *P. mirabilis* in UC inflammation, we used a dextran sulfate sodium (DSS)-induced colitis animal model. DSS, a chemical agent known to increase epithelial permeability and thereby elevate the risk of enteric infections, was administered by adding it to the drinking water at 2% concentration for 1 week. On the fifth and sixth days after DSS administration, oral gavage of 1 × 10^9^ CFU *P. mirabilis* or 100 μl PBS (control) was performed. In contrast to the control, the animals that received *P. mirabilis* exhibited a significant loss of body weights, and more severe diarrhea and fecal hemorrhage, while the control group did not exhibit significant weight loss, diarrhea, or fecal hemorrhage (Figure 2A). Quantification revealed that the disease activity index of *P. mirabilis* group was significantly higher than the control (Figure 2B). Consistently, the colon length of the *P. mirabilis* group was shorter than that of the control (*P. mirabilis* group: 6.0 ± 0.2 cm vs. Ctr group: 6.8 ± 0.5 cm, *P* < 0.01; Figures 2C, D). Histological examination of *P. mirabilis* group showed more severe inflammation, and other UC-characteristic pathological alterations including distortion of crypt architecture, crypt abscesses, immune cell infiltration, micro-abscesses in the lamina propria, crypt shortening, mucin depletion, lymphoid aggregates, erosion, and ulceration (Figures 2E, F). We measured the levels of classical inflammatory cytokines and chemokines upregulated in UC mucosa, such as *tnfa, il6, il1b, il12b, ifng, il23a, il17a, il22, cxcl1* and *ccl2*. Notably, the cytokine profiling showed that the mRNA level of *cxcl1*, a key chemokine responsible for recruiting neutrophils, was significantly upregulated (Figure 2G). Additionally, the mRNA levels of cytokines associated with UC inflammation such as *il1b, il6, il12b, ifng, il17a*, were also upregulated (Figure 2G). Thus, these results clearly suggest that introduction of *P. mirabilis* significantly intensifies the UC inflammation.

### 3.4 *P. mirabilis* infection reduced mucus layer thickness in DSS colitis inflammation

**Figure 3:**
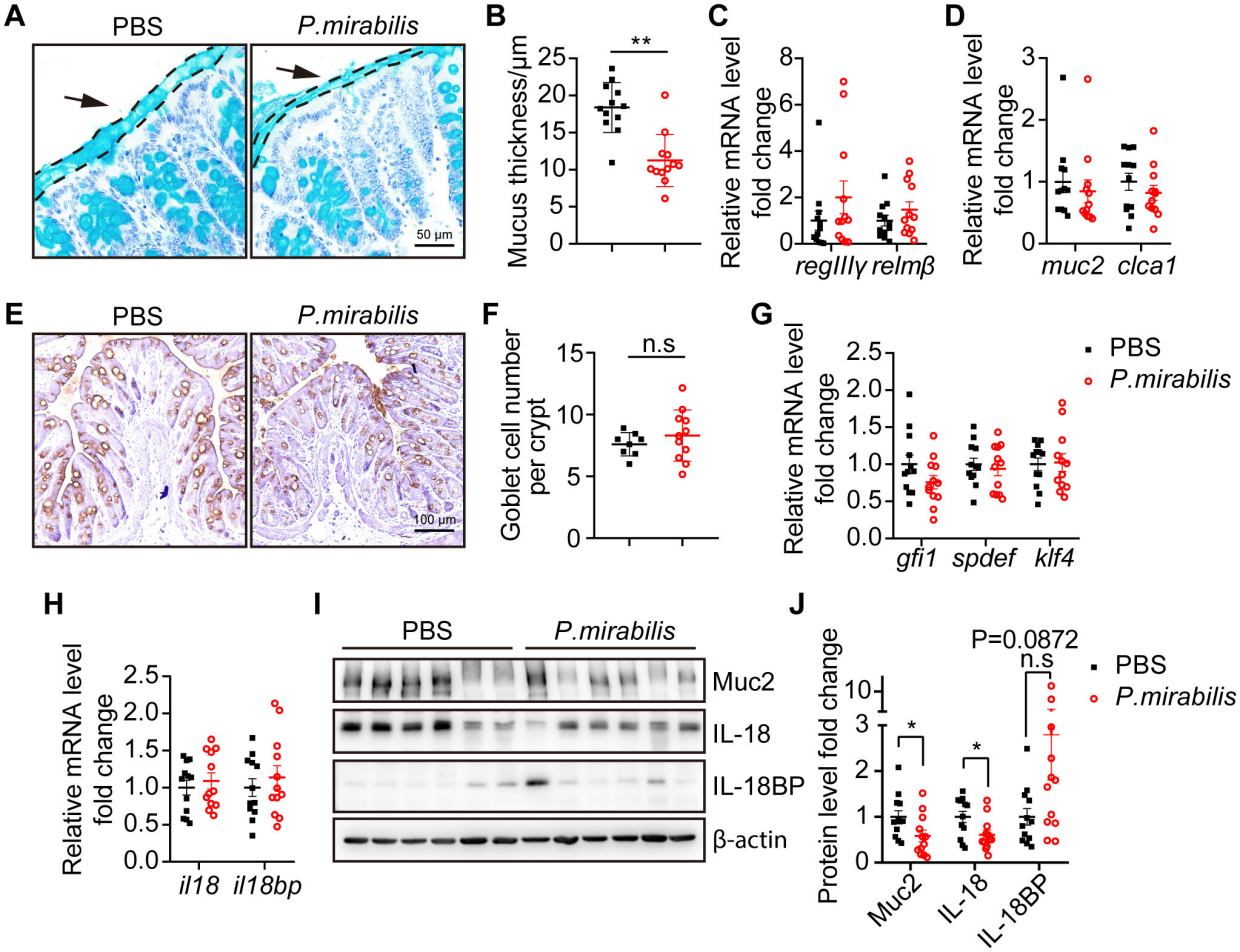
*P. mirabilis* infection impaired mucus layer formation by inhibiting IL-18 protein. D-colon tissue from mice received PBS (*n* = 12) or *P. mirabilis* (*n* = 12) during 2% DSS treatment were collected for mucus layer analysis. **(A)** PAS-AB staining of colon sections fixed with methacarn. Arrows and dashed lines showed the boundary of mucus layer. Scale bar, 50 μm. **(B)** Quantification of mucus thickness. Each dot represents the mean goblet cell count per crypt for each mouse. **(C)** Relative mRNA levels of antimicrobial peptide. **(D)** Relative mRNA levels of goblet cell markers. **(E)** Immunohistochemistry staining of colon section with an anti-Muc2 primary antibody. Scale bar, 100 μm. **(F)** Quantification of goblet cell number. Each dot represent mean of goblet cell number per crypt in each mice. **(G)** Relative mRNA levels of transcription factors associated goblet cell differentiation. **(H)** Relative mRNA levels of *il18* and *il18bp*. **(I)** Western blot analysis relative expression of Muc2, IL-18 and IL-18BP in colon tissue. **(J)** Quantification of relative protein level fold change. Data was normalized by mean of PBS group. **(B–D, F–H, J)** Represent as mean ± SEM, statistic with unpaired 2-tailed *t* test. n.s. > 0.05, *P < 0.05, **P < 0.01. Black square, *P. mirabilis* group; red circle, PBS group.

The mucus layer serves as the first line of defense in the colonic mucosal barrier against microbial invasion. In this study, we measured the mucus layer thickness in the colon using PAS-AB staining. The results showed that the mucus layer over the epithelial barrier in the *P. mirabilis* group was significantly thinner than that of the control group (*P. mirabilis* group: 11.23 ± 1.01 μm vs. control group: 18.38 ± 0.97 μm, *P* < 0.01; Figures 3A, B). The relative mRNA levels of the antimicrobial peptide *regIII*γ and *relm*β were upregulated in the *P. mirabilis* group (Figure 3C), suggesting an increased risk of microbial translocation and immune response activation (Vaishnava et al., 2011). Mucin 2 (Muc2), a high-molecular-weight glycoprotein produced by goblet cells, is the main component of the mucus layer. We measured the mRNA levels of *muc2* and *clca1* (a secreted metalloprotease produced by goblet cells) and found no significant difference between the *P. mirabilis* and control groups (Figure 3D). Immunohistochemistry with an anti-Muc2 antibody showed comparable numbers of Muc2^+^ goblet cells in the distal colon of the *P. mirabilis* group (Figures 3E, F). The relative mRNA levels of goblet cell differentiation factors *gfi1, spdef*, and *klf4*, were also similar between the two groups (Figure 3G). However, immunoblotting revealed a significant decrease in Muc2 protein levels in *P. mirabilis*-infected colon tissues compared to the control group (Figures 3I, J). Thus, *P. mirabilis* infection impairs the mucus layer, and thereby allows pathogens to access colonic epithelium and exacerbates enteric infections. Since *P. mirabilis* did not cause abnormal goblet cell depletion or differentiation, the thinner mucus layer observed might result from impaired mucin production.

### 3.5 *P. mirabilis* infection impaired IL-18-dependent mucin production

It has been reported that IL-18 plays a role in homeostatic intestinal barrier maintenance and mucin production (Nowarski et al., 2015; Pu et al., 2019; Chiang et al., 2022), while IL-18 binding protein (IL-18BP) serves as the decoy receptor of IL-18 for IL-18 equilibrium (Novick et al., 1999; Dinarello et al., 2013). To test whether *P. mirabilis* infection can inhibit IL-18 expression and thereby impair mucin production, we measured the mRNA levels of *il18* and *il18bp*, and found no significant difference between the *P. mirabilis* and control groups (Figure 3H). However, Western blot analysis revealed a significant decrease in IL-18 protein in the *P. mirabilis* group (Figures 3I, J), whereas the IL-18BP protein levels was upregulated in the colon tissue (Figures 3I, J). This result indicates that *P. mirabilis* infection reduced mucin production through downregulation of IL-18 protein expression in inflamed colons.

### 3.6 Bacteriophage treatment relieved *P. mirabilis* infected DSS colitis

**Figure 4:**
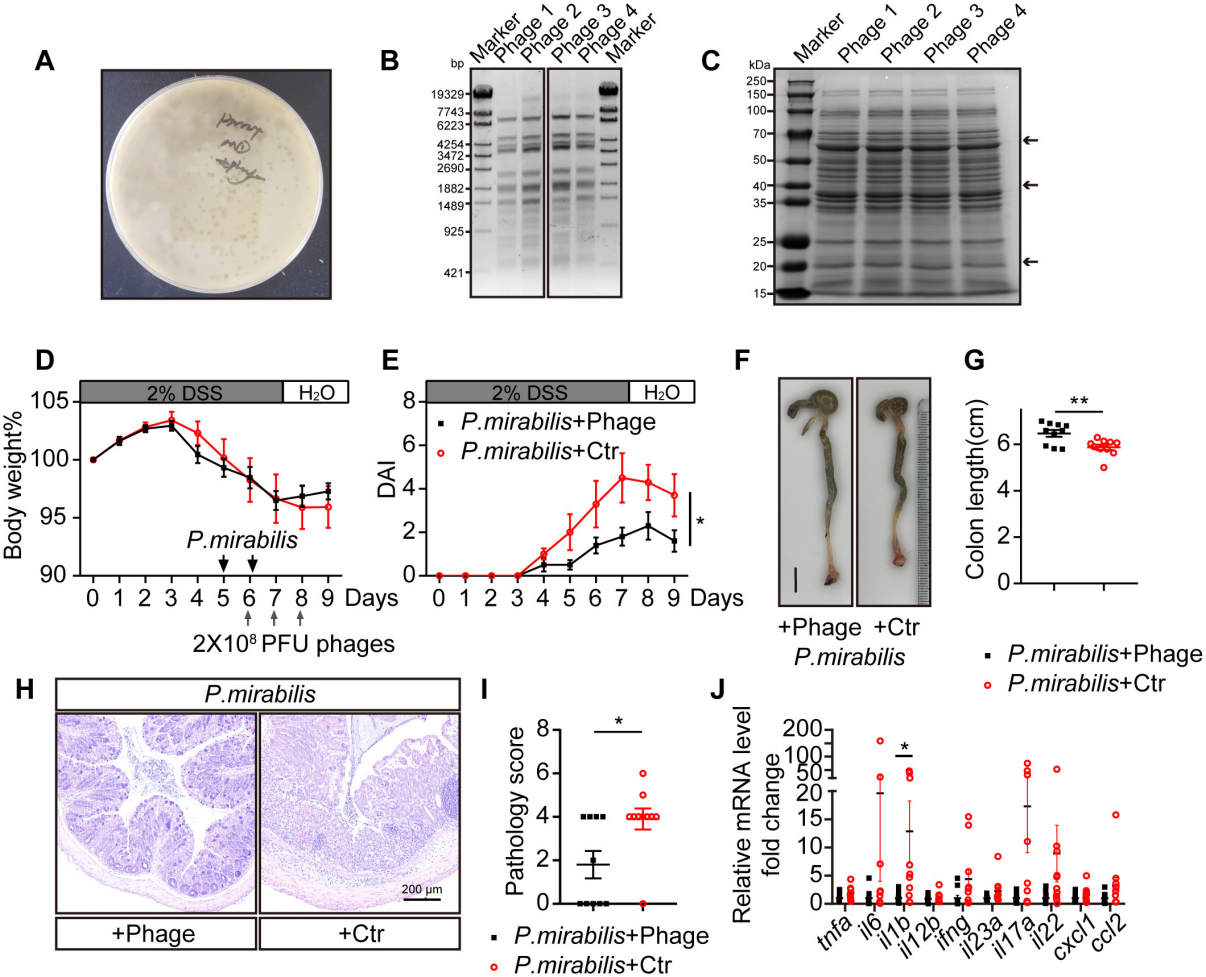
Phage cocktail targeting *P. mirabilis* protected mice from severe colitis symptom induced by *P. mirabilis* infection. Lytic phages targeting *P. mirabilis* were isolated and used as phage therapy to treat *P. mirabilis* inducing colitis symptom. 2 × 10^8^ PFU phage cocktail or 200 μL control buffer was gavaged to mice at gray arrow indicated time point. *P. mirabilis*+Phage group (*n* = 10); *P. mirabilis* + Ctr group (*n* = 10). **(A)** Representative *P. mirabilis* targeting phage formed bacteriophage plaque on double layer LB agar. **(B)** DNA pattern of four *P. mirabilis* targeting phage digested with *EcoR V* restriction enzyme. **(C)** Protein pattern of four *P. mirabilis* targeting phage separated by SDS-PAGE, staining with coomassie brilliant blue. Black arrows indicate distinctive bands. **(D)** Body weight change. **(E)** Daily disease activity index. **(F)** Representative picture of colon. Scale bar, 1 cm. **(G)** Colon length. **(H)** H&E staining of colon section. Scale bar, 200 μm. **(I)** Pathology score of colon. **(J)** Relative cytokine mRNA level of colon, normalized by *P. mirabilis* + Phage group. **(D, E, G, I, J)** Represent as mean ± SEM. n.s > 0.05, *P < 0.05, **P < 0.01. **(D, E)** Two-way *ANOVA* with Geisser-Greenhouse correction. **(G, I, J)** Unpaired 2-tailed *t* test. Black square, *P. mirabilis* + Phage group; red circle, *P. mirabilis* + Ctr group.

To explore a safe and effective therapeutic method for UC, we screened the natural wastewater and obtained *P. mirabilis*-targeting lytic bacteriophages (phages) (Figure 4A). *EcoR V* restriction enzyme digestion of phage DNA and SDS-PAGE separation of protein patterns indicated four different types of phages (Figures 4B, C). These phages were combined into a phage cocktail to treat *P. mirabilis*-infection in mice. For the animals received *P. mirabilis* and DSS as described above, 2 × 10^8^ PFU of the phage cocktail was orally administered for 3 consecutive days. Although there was no significant difference in body weight loss (Figure 4D), the animals treated with the phage cocktail showed significantly reduced colitis symptoms, including diarrhea and bloody stools (Figure 4E). Compared to the control group, the phage-treated group had longer colon lengths (phage group: 6.5 ± 0.1 cm vs. control group: 5.9 ± 0.1 cm, *P* < 0.01; Figures 4F, G). Pathological analysis showed that the phage cocktail significantly protected against mucosal ulceration and inflammatory cell infiltration (Figure 4H), with a lower pathology score (Figure 4I). The mRNA levels of inflammatory cytokines such as *il1b, tnfa, ifng*, and *il17a* were downregulated after phage therapy (Figure 4J). Thus, the *P. mirabilis*-targeting phage cocktail could ameliorate enteric inflammation.

### 3.7 Eliminating *P. mirabilis* restored mucus layer thickness *in vivo*

**Figure 5:**
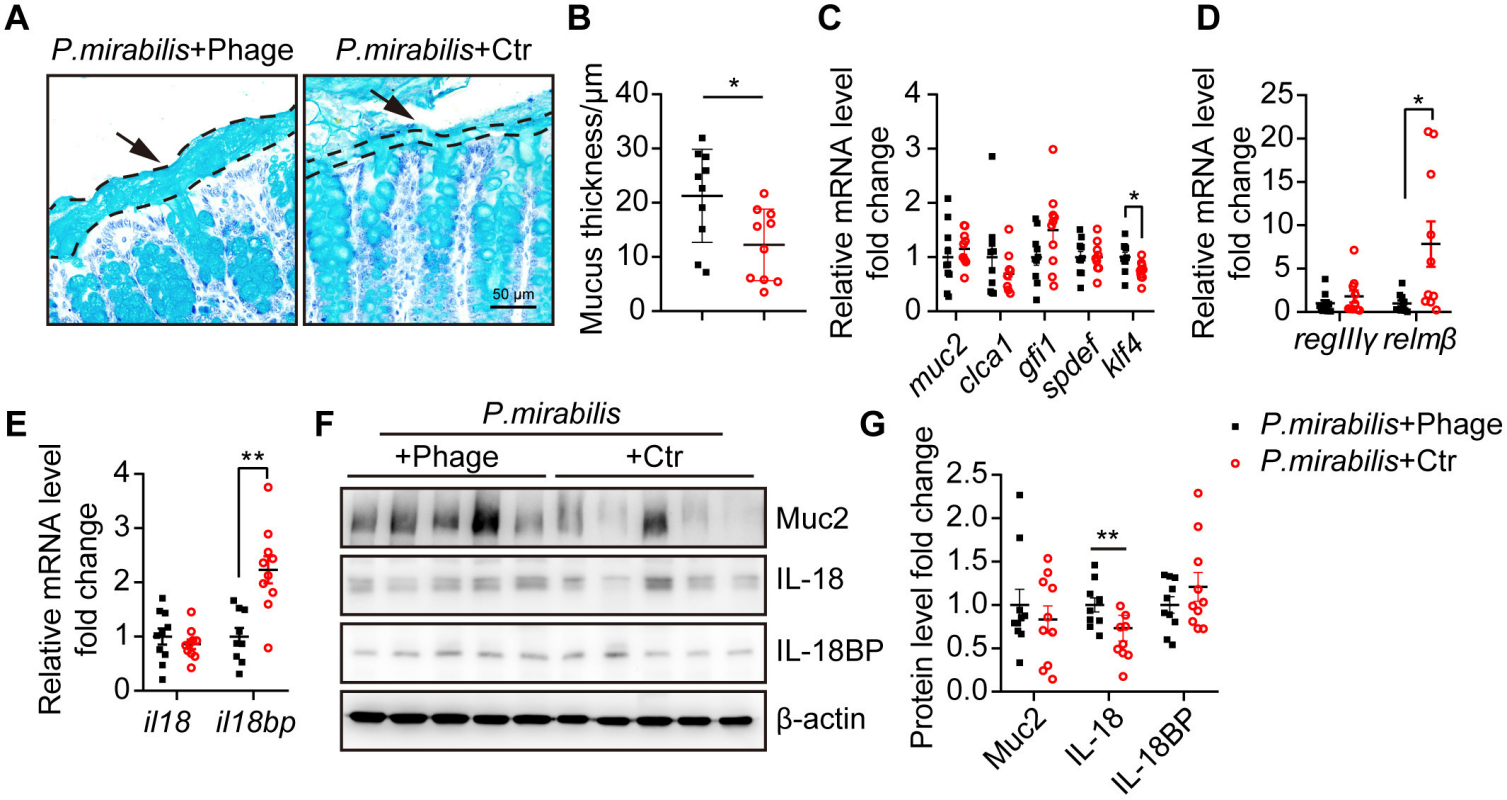
Phage cocktail targeting *P. mirabilis* restored mucus layer thickness. Distal colon tissue from mice received *P. mirabilis* + Phage (*n* = 10) or *P. mirabilis* + Ctr (*n* = 10) during 2% DSS treatment were collected for mucus layer analysis. **(A)** PAS-AB staining of colon sections fixed with methacarn. Arrows and dashed lines showed the boundary of mucus layer. Scale bar, 50 μm. **(B)** Quantification of mucus thickness. Each dot represents the mean of mucus layer thickness from at least 10 view of each colon section. **(C)** Relative mRNA level of goblet cell markers and differentiation transcription factors. **(D)** Relative mRNA level of antimicrobial peptide. **(E)** Relative mRNA level of *il18* and *il18bp*. **(F)** Western blot analysis relative expression of Muc2, IL-18 and IL-18BP. β-actin was used as an internal reference. **(G)** Relative protein level fold change. Data was normalized by mean of *P. mirabilis* + Phage group. **(B–E, G)** Represent as mean ± SEM, Statistic with unpaired 2-tailed *t* test. n.s. > 0.05, ^*^P < 0.05, ^**^P < 0.01. Black square, *P. mirabilis* + Phage group; red circle, *P. mirabilis* + Ctr group.

As observed above, a thinner mucus layer is a typical feature in the *P. mirabilis*-infected DSS colitis mouse model. To determine whether eliminating *P. mirabilis* with a phage cocktail could restore the mucus layer, we measured the thickness of the mucus layer of the treated mice. In contrast to the control group, the phage cocktail significantly restored the mucus layer (phage group: 21.29 ± 2.72 μm vs. control group: 12.26 ± 2.09 μm, *P* < 0.05) (Figures 5A, B). The relative mRNA levels of *muc2* and *clca1* in the phage-treated and control groups were comparable, as were those of goblet cell differentiation factors *gfi1* and *spdef*. However, phage treatment significantly upregulated the transcription of *klf4* (Figure 5C), a factor that regulates goblet cell maturation (Katz et al., 2002). Notably, phage treatment significantly decreased *relm*β mRNA level (Figure 5D), suggesting that phage treatment reduced the translocation of invasive bacteria to the mucosal epithelium. The mRNA levels of *il18bp*, but not *il18*, were significantly inhibited after phage treatment (Figure 5E). Western blot analysis confirmed the upregulation of IL-18 protein following phage treatment (Figures 5F, G). Therefore, the phage cocktail successfully restored the mucus layer and IL-18 protein levels in *P. mirabilis*-infected DSS colitis.

## 4 Discussion

*P. mirabilis* is a facultative anaerobic, gram-negative bacterium belonging to the *Enterobacteriaceae* family and serves as a commensal gastrointestinal bacterium with low abundance. There is report showing that *P. mirabilis* is associated with ~10% of urinary tract infections (UTIs), particularly in catheter-associated UTIs (CAUTIs) (Armbruster and Mobley, 2012). In the case of UC, although increased *Gammaproteobacteria, Enterobacteriaceae*, and sulfate-reducing *Deltaproteobacteria* in the mucosa have been populated by mega 16S rRNA sequencing (Roediger et al., 1997; Frank et al., 2007; Dai et al., 2021), which species or strains of these bacteria are functionally associated with UC inflammation remains unclear because 16S rRNA sequences cannot identify bacteria at the species or strain level. Until recently, *P. mirabilis* has been identified as a potential pathogen in CD recurrence and disease inflammation (Mondot et al., 2016; Wright et al., 2017). We here detected the frequent presence of fecal *P. mirabilis* in UC patients also. Among 41 UC patients, 65.9% were *P. mirabilis*-positive, which was significantly higher than that of healthy groups (3.3%) and IFC patients (6.3%). Further analysis indicates that this distribution is indifferent to age or gender. Thus, *P. mirabilis* expansion may be associate with UC gut inflammation, rather than representing a non-specific alteration of the microbiome. The observations from other investigators who use different detection methods for *P. mirabilis* also support this conclusion (Müller, 1986; Kanareykina et al., 1987; Khorsand et al., 2022; Kushkevych et al., 2024).

Based on our observation, the role of *P. mirabilis* in UC pathology may be characterized by exacerbating colitis symptoms, histological changes, and elevated levels of inflammatory cytokines. As we know, altered inflammatory cytokines essentially evolve in enteric Inflammatory of UC or IBD, e.g., TNFα, IL-6, IL-1β, IL-12, IL-23, IFNγ, and IL-17 are elevated and thereby regulate inflammation responses (de Souza and Fiocchi, 2016; Nakase et al., 2022). IL-6 promotes macrophage activation and T cell differentiation, including the differentiation of IL-17-producing Th17 cells (Baumgart and Sandborn, 2012). IL-17 further induces the production of other inflammatory mediators, such as IL-6, TNFα, and IL-1β, which contribute to the cell death of intestinal epithelial cells, thereby impairing intestinal integrity. Additionally, IL-17A induces the production of chemokines like CXCL1, which recruit neutrophils and other immune cells to the inflamed mucosa, leading to colonic ulceration and crypt abscesses (Neurath, 2014). We observed that the infection of *P. mirabilis* infection primarily activated IL-6/IL-17A axis and simultaneously upregulated the expression of TNFα, IL-1β, IFNγ, and CXCL1, but the expression of IL-23a were not intensified. This cytokine profile seems different from that of CD patients (Seishima et al., 2019) and suggests that *P. mirabilis* displays distinct roles in UC and CD through different virulence factors. Collectively, the upregulation of these cytokines suggests that *P. mirabilis* exacerbates UC pathology by enhancing pro-inflammatory signaling pathways and disrupting immune homeostasis.

Several virulence factors of *P. mirabilis* for enteric inflammation have been discovered such as protease ZapA, hemolysins, endotoxin, flagellins, and urease. Protease ZapA functions to help *P. mirabilis* evasion through proteolytic digestion of innate immune antimicrobial peptide (Walker et al., 1999; Belas et al., 2004); Hemolysin activates NOD-like receptor protein 3 (NLRP3) inflammasome and IL-1β release, thereby intensifying gut inflammation (Seo et al., 2015); Endotoxins and flagellins activate downstream inflammatory pathways (Hayashi et al., 2001; Akira et al., 2006). According to our observations that *P. mirabilis* evokes multiple responses associated with these virulence factors, we guess that multiple virulence factors of *P. mirabilis* may synergistically contribute to the pathology of UC. In addition, such pathogenic or opportunistic bacteria as *P. mirabilis* usually express urease to establish a alkaline environment for their competitive expansion (Kamada et al., 2013; Winter et al., 2013), and the expressed urease has additional activities toward inflammation induction via production of reactive oxygen species (ROS) and cytokines such as IL-1β and TNFα (Grahl et al., 2021). The urease produced by *P. mirabilis* might thus contribute to UC inflammation also.

Several pathobionts have been isolated from IBD patients, and these isolates exacerbate IBD inflammation through various mechanisms (Mirsepasi-Lauridsen et al., 2020; Yang et al., 2020; Baumgartner et al., 2022). Among these, adherent-invasive *Escherichia coli* is the most well-established pathobiont of IBD. AIEC adheres to and invades intestinal epithelial cells, replicates within macrophages, disrupts the epithelial barrier, triggers inflammation, and contributes to dysbiosis (Boudeau et al., 1999; Glasser et al., 2001; Chassaing et al., 2011; Govindarajan et al., 2020). *Clostridium innocuum* isolated from mesenteric adipose tissue in CD patients exacerbates the creeping fat phenotype by metabolizing lipids, beta-hydroxybutyrate, and detoxifying oxygen (Ha et al., 2020). *Atopobium parvulum* intensifies IBD severity by disrupting the gut environment through its metabolic products (Mottawea et al., 2016). Proteases-producing *Bacteroides vulgatus* and candidalysin producing *Candida albicans* are also implicated in UC pathogenesis (Li et al., 2022; Mills et al., 2022). Our data show that *P. mirabilis* aggravates UC inflammation by impairing the mucus layer, a novel mechanism for IBD pathobionts. It has been reported that *P. mirabilis* increases intestinal inflammation by promoting cytokine production and activating NLRP3-caspase1 signaling (Seo et al., 2015; Zhang et al., 2021). Our data revealed that the reduction of the mucin layer mediated by *P. mirabilis* may also contribute to IBD inflammation. On the other side, it should be noted that 34.1% of UC patient stool specimens were *P. mirabilis*-negative. This means other pathobionts may be involved in UC pathological process also.

Patients with active UC often exhibit depleted colonic goblet cells and a compromised mucus barrier (Johansson et al., 2014). NLRP3 can increase mucus production from goblet cells to protect the mucosa through the IL-18-dependent pathway (Engler et al., 2015). IL-18, an IL-1 family cytokine, is activated by inflammasome-caspase1-cascade and constitutively expressed in the intestinal epithelium and macrophages, and was first described as a Th1 immune-skewing cytokine that induces IFNγ production (Fantuzzi et al., 1999). In contrast to IL-18, IL-18BP is produced by a broad range of hematopoietic and non-hematopoietic cell types (Harel et al., 2020). Th1 cytokines such as IFNγ and IL-18 are capable to induce IL18-BP expression (Veenstra et al., 2002). Given the ability of urease and hemolysins to activate inflammasome-mediated caspase-1, these two virulence factors may cooperatively contribute to the induction of IL-18BP through the IL-18-IFN-γ-IL-18BP negative feedback loop during *P. mirabilis* infection. In the plasma of IBD patients, both UC and CD patients have higher levels of IL-18BP, while free, unbound IL-18 is elevated in CD patients rather than healthy populations and UC patients (Ludwiczek et al., 2005). Since IL-18BP can bind IL-18 with high affinity and reduce its availability (Dinarello et al., 2013), our result suggests that a low level of IL-18 protein may result from the upregulation of IL-18BP of *P. mirabilis* infection and the resultant reduction of IL-18 subsequently leads to mucus layer reduction. The mucus layer consists of a loose outer layer and a dense inner layer. The loose layer provides a habitat for commensal microbes, while the dense layer is essential for preventing direct contacting between the intestinal epithelium and microbes (Johansson et al., 2008; Martens et al., 2018). Certain anti-inflammatory commensal bacteria, such as *Akkermansia muciniphila*, use mucins and glycoproteins in mucus layer as a nutrient source and are reduced in UC (Zheng et al., 2022). Thus, the *P. mirabilis*-induced compromised mucus layer may not only impair barrier function but also cause dysbiosis by inhibiting commensal microbes, which results in further aggravating inflammation. However, the mechanisms by which virulence factors of *P. mirabilis* regulate the IL-18/IL-18BP equilibrium require further investigation.

Based on our observations, the role of *P. mirabilis* in UC progression is to primarily intensify enteric inflammation secondary to UC. Thus, *P. mirabilis* may be considered a pathobiont in UC. Given the essential role of *P. mirabilis* in UC progression, the elimination of this bacterium may be beneficial for UC therapy. Antibiotics and faecal microbiota transplantation (FMT) are used as therapies targeting the microbiota in UC (Caruso et al., 2020). However, antibiotic resistance has become a major global health challenge that significantly impacts clinical UC treatment (Larsson and Flach, 2022). Unfortunately, *P. mirabilis* is inherently resistant to several antibiotics, including polymyxins (e.g., colistin), nitrofurans, tigecycline, and tetracycline (Rózalski et al., 1997; Olaitan et al., 2014; Qin et al., 2015). Moreover, acquired resistance is a major concern, with *P. mirabilis* acquiring resistance genes through conjugation, plasmids, and mobile genetic elements such as *Salmonella* genomic island 1 (SGI1), which confers resistance to streptomycin, trimethoprim, tetracycline, sulfonamides, chloramphenicol, fluoroquinolones, and broad-spectrum β-lactam antibiotics (Qin et al., 2015). The use of antibiotics could increase the risk of developing UC and other enteric inflammations, limiting the effectiveness of antibiotic treatments (Faye et al., 2023; Duan et al., 2024). On the other hand, the efficacy of FMT in treating UC is also limited and inconsistent, with only 30% of UC patients achieving clinical remission (Paramsothy et al., 2017; Levy and Allegretti, 2019). As an alternative therapy, bacteriophages have shown remarkable effectiveness in eliminating target bacteria and relieving infectious diseases efficiently (Duan et al., 2022; Ichikawa et al., 2023). As bacteriophages have high specificity for targeting pathobionts, use of phages will not disturb the homeostasis gut bacteria (Correa et al., 2021). Typically, a phage cocktail is required to reduce the risk of bacterial resistance (Egido et al., 2022). We isolated four phages targeting *P. mirabilis*, and found that a bacterial phage cocktail could effectively kill *P. mirabilis* both *in vitro* and *in vivo*, thereby attenuating UC symptoms. Thus, we propose that phage therapy may be a promising treatment for UC. However, due to the complexity of the gut microbiota and the limited understanding of *P. mirabilis* and other pathobionts in UC, more studies are needed to verify the clinical outcomes of bacteriophage therapy.

In conclusion, our work demonstrates that *P. mirabilis* is a pathobiont that inhibits the mucus barrier and exacerbates UC inflammation. Phage therapy targeting *P. mirabilis* may be beneficial for UC treatment.

## Data Availability

The original contributions presented in the study are included in the article/Supplementary material, further inquiries can be directed to the corresponding authors.
