# Supplementary material for: Proteus mirabilis exacerbates ulcerative colitis by inhibiting mucin production
Source: Front Microbiol. 2025 Mar 25;16:1556953. doi: 10.3389/fmicb.2025.1556953 (PMC11975560; doi:10.3389/fmicb.2025.1556953)
Supplement: Supplementary file 1 [file Table_1.docx]

**SUPPLEMENTARY MATERIALS**

**Table S1. qPCR primer list**

| **Gene** | **Forward 5' to 3'** | **Reverse 5' to 3'** |
| --- | --- | --- |
| *Ifng* | GATGCATTCATGAGTATTGCCAAGT | GTGGACCACTCGGATGAGCTC |
| *Tnfa* | CTGAACTTCGGGGTGATCGG | GGCTTGTCACTCGAATTTTGAGA |
| *Il6* | TAGTCCTTCCTACCCCAATTTCC | TTGGTCCTTAGCCACTCCTTC |
| *Il17a* | CTCCAGAAGGCCCTCAGACTAC | AGCTTTCCCTCCGCATTGACACAG |
| *Il23a* | AATAATGTGCCCCGTATCCAGT | GCTCCCCTTTGAAGATGTCAG |
| *Il22* | TTGAGGTGTCCAACTTCCAGCA | AGCCGGACGTCTGTGTTGTTA |
| *Il1b* | AAAGATGAAGGGCTGCTTCC | TTCTCCACAGCCACAATGAG |
| *Il12b* | GTCCTCAGAAGCTAACCATCTCC | CCAGAGCCTATGACTCCATGTC |
| *Ccl2* | TTAAAAACCTGGATCGGAACCAA | GCATTAGCTTCAGATTTACGGGT |
| *Cxcl1* | ACTGCACCCAAACCGAAGTC | TGGGGACACCTTTTAGCATCTT |
| *Gapdh* | TCACCACCATGGAGAAGGC | GCTAAGCAGTTGGTGGTGCA |
| *Gfi1* | AGAAGGCGCACAGCTATCAC | GGCTCCATTTTCGACTCGC |
| *Spdef* | AAGGCAGCATCAGGAGCAATG | CTGTCAATGACGGGACACTG |
| *Klf4* | GTGCCCCGACTAACCGTTG | GTCGTTGAACTCCTCGGTCT |
| *RegIIIγ* | TCAGGTGCAAGGTGAAGTTG | GGCCACTGTTACCACTGCTT |
| *Relmβ* | AAGCCTACACTGTGTTTCCTTTT | GCTTCCTTGATCCTTTGATCCAC |
| *Muc2* | ATGCCCACCTCCTCAAAGAC | GTAGTTTCCGTTGGAACAGTGAA |
| *Clca1* | CTGTCTTCCTCTTGATCCTCCA | CGTGGTCTATGGCGATGACG |
| *Il18* | GACTCTTGCGTCAACTTCAAGG | CAGGCTGTCTTTTGTCAACGA |
| *Il18bp* | GAGGGCCACACAAGTCGC | GCTGGGCCAGAATGATGTGA |
